# Historical Aspects and Relevance of the Human Coronary Collateral Circulation

**DOI:** 10.2174/1573403X113099990028

**Published:** 2014-02

**Authors:** Christian Seiler, Pascal Meier

**Affiliations:** 1 Department of Cardiology, University Hospital, Bern, Switzerland;; 2 University College London, Division of Cardiology, London, UK

**Keywords:** Coronary artery disease, coronary circulation, collateral circulation.

## Abstract

In 1669, anastomoses between the right and left coronary artery were first documented by Richard Lower of Amsterdam. Using post-mortem imaging, a debate followed on the existence of *structural* inter-coronary anastomoses, which was not resolved before the first half of the 20ieth century in case of the presence of coronary artery disease (CAD), and not before the early 1960ies in case of the normal human coronary circulation by William Fulton. *Functional* coronary collateral measurements during coronary interventions were first performed only in the 1970ies, respectively in the early 1980ies. In humans, the existence of *functional* coronary collaterals in the absence of CAD has not been documented before 2003.

Though the coronary collateral circulation has been recognized as an alternative source of blood supply to ischemic myocardium, its prognostic significance for the CAD population as a whole has been controversial until recently. The debate was due to different populations examined (acute versus chronic CAD, varying severity of CAD), to variable definitions of the term “prognosis”, to insufficient statistical power of the investigation with rare occurrence of prognostic endpoints, to short duration of follow-up and to blunt instruments employed for collateral assessment. Individually, it has been acknowledged that a well functioning collateral supply to a myocardial area at risk for necrosis reduces infarct size, preserves ventricular function, prevents ventricular remodelling and aneurysm formation. Collectively, evidence has accumulated only recently that an extensive coronary collateral circulation is a beneficial prognosticator quoad vitam. In a recent meta-analysis on the topic, the risk ratio to die from any cause for high vs low or absent collateralization in patients with subacute myocardial infarction was 0.53 (95% confidence interval 0.15–1.92; p=0.335), and for patients with acute myocardial infarction, it was 0.63 (95% confidence interval 0.29–1.39; p=0.257)¸ the relative risk to die from any cause for well vs poorly developed collaterals in patients with stable CAD was 0.59 (95% confidence interval 0.39–0.89), p=0.012.

## HISTORICAL ASPECTS

**Fig. (1):**
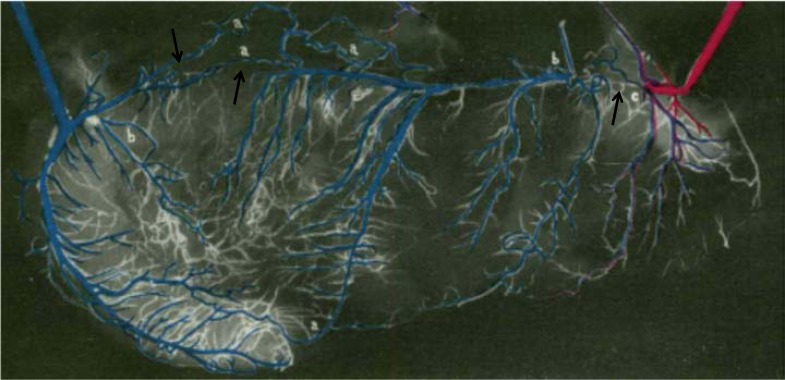
Coloured coronary angiogram of a human “unrolled” heart with multiple coronary artery occlusions (→) and extensive inter-arterial coronary anastomoses. Coronary collateral vessels are recognizable by grossly dissectible channels between two arteries (a), by injection mass of any colour distal to a complete coronary artery occlusion (b), and by admixture of colours of injection mass (c) 6.

**Fig. (2):**
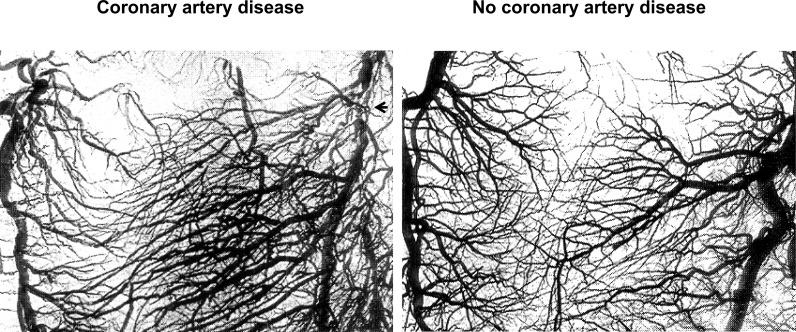
Image of numerous interventricular anastomoses via septal branches of the left anterior descending coronary artery (LAD) in a patient with cor-onary artery disease (left side panel; chronic total occlusion of the LAD →), and in an individual with normal heart (right side panel). Small and medium sized collateral vessels (50-200mm in diameter) can be traced between the LAD on the right side of the images and the ramus interven-tricularis posterior of the right coronary artery on the left side. Image magnification: 2x.

Angina pectoris as a symptom was unknown before the late 18th century [1]. For a long time, physicians thought that angina pectoris was very often fatal [2]. On the other hand, evolving tolerance to effort-induced chest pain which could even “cure” it was described by William Heberden [3]. Hence in 1669, the first description of anastomoses between the right and left coronary arteries by Richard Lower of Amsterdam had occurred independently of the discovery of the symptom of angina pectoris [4]. In 1757, the Swiss anatomist Albrecht von Haller also described structural coronary anastomoses [5]. The first anatomic observations of anastomoses were possibly made in coronary arteries unaffected by severe obstructions, because coronary artery disease (CAD) used to be much less prevalent than today. Using a multitude of different post-mortem imaging techniques, a controversy on the existence of structural inter-coronary anastomoses followed their first description, which was not resolved in their favour before the mid 20ieth century for patients with CAD [6, 7] (Fig. **1**), and not before the 1960ies for normal human hearts by William Fulton [8, 9] (Fig. **2**). Since James Herrik’s clinico-pathologic description in 1912 of a patient surviving sudden thrombotic coronary obstruction in the presence of inter-coronary anastomoses [2], it became evident that the collateral circulation is an important determinant of the rate and extent of myocardial cell death. However, actual *in vivo* functional coronary collateral measurements in patients undergoing bypass surgery or percutaneous coronary intervention (PCI) were first performed only in the 1970ies, respectively in the early 1980ies [10, 11]. The existence of *in vivo* functional collaterals in the atherosclerotic coronary obstructions has not been documented until 2003 [12].

## INDIVIDUAL RELEVANCE

Since the time when factors such as time of occlusion, myocardial area at risk for infarction, absence of collateral supply, absence of ischemic preconditioning, myocardial oxygen consumption during occlusion were described as determinants of myocardial infarct size [13, 14], it has become evident that collateral flow is one of the most important factors of the rate and extent of myocardial cell death. However individually, well developed coronary collaterals may also manifest negative aside from beneficial effects.

## BENEFICIAL EFFECT OF COLLATERALS ON MYOCARDIAL SALVAGE

**Fig. (3):**
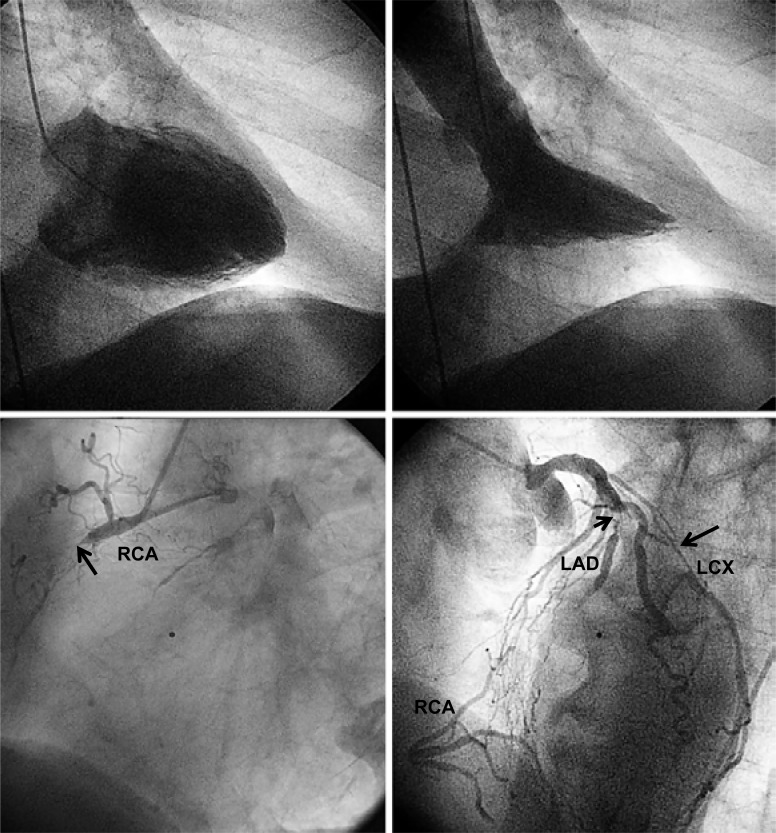
Normal left ventricular angiogram (upper panels) in a patient with chronic proximal occlusion (→) of all three major coronary arteries (lower panels) providing evidence for the myocardial-salvaging effect of coronary collaterals. The only non-occluded vessel is an intermediary branch. RCA: right coronary artery; left anterior descending coronary artery: LAD; left circumflex coronary artery: LCX.

**Fig. (4):**
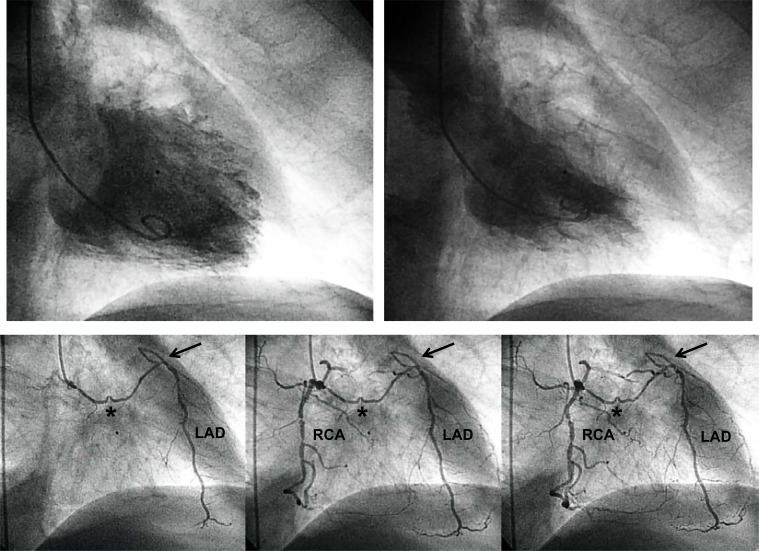
Normal left ventricular angiogram (upper panels) in a patient with chronic occlusion (→) of the proximal left anterior descending coronary artery (LAD; lower panels, antero-posterior cranial view). Contrast injection into the right coronary artery (RCA) with imaging at first of the conus branch collateral artery to the LAD (*). A second, atrial collateral artery between RCA and LAD (middle and right lower panel) fills with contrast from both coronary arteries, thus illustrating competing collateral flow between RCA and LAD.

**Fig. (5):**
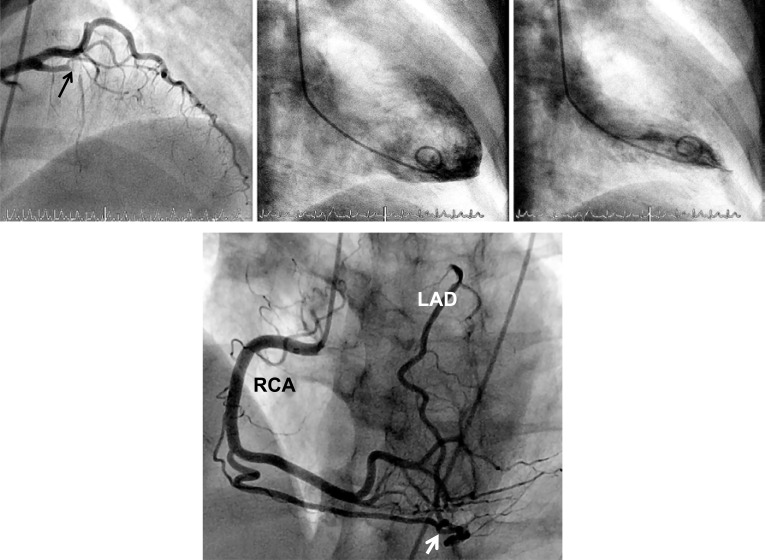
Coronary angiogram (left anterior oblique cranial view; left panel) with chronic occlusion of the left anterior descending artery at the first diagonal and first septal branch (→) and a normal left ventricular angiogram (middle and left panel) exemplifying another case of saving myocardial function by well developed collaterals. Lower panel: Coronary angiogram (slight left anterior oblique cranial view) with contrast injection into the right coronary artery (RCA) and complete filling of the proximally occluded left anterior descending artery (LAD) via a large branch collateral artery (→).

**Fig. (6):**
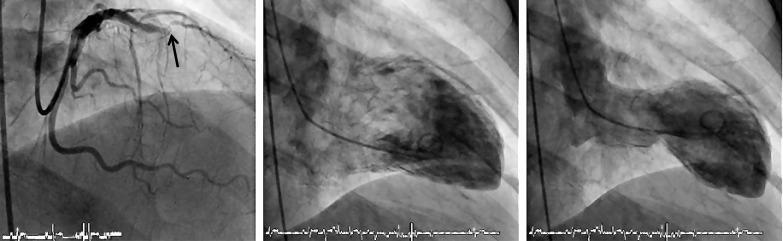
Coronary angiogram (antero-posterior view; left panel) with occlusion of the left anterior descending artery (LAD) directly distal to the second diagonal and second septal branch (→). In the absence of collateral supply to the occluded LAD, the left ventricular angiogram (middle and left panel) reveals an extensive akinesia of the antero-apical wall.

**Fig. (7):**
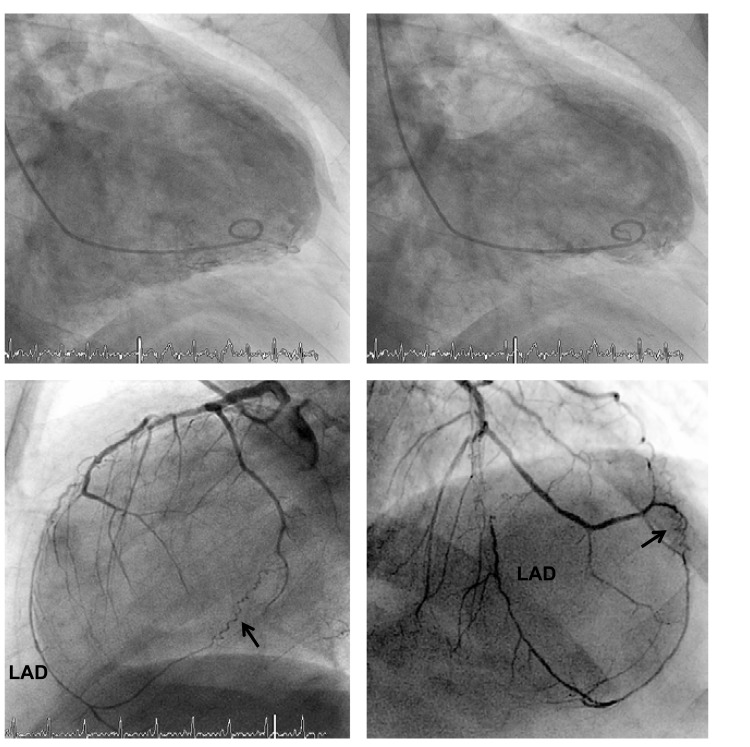
Left ventricular angiogram (upper panels) with extensive antero-apical wall akinesia in a case with a well developed branch collateral artery (→) between the first diagonal branch of the left anterior descending artery (LAD) and the occluded LAD (lower panels; left panel: lateral view; right panel: antero-posterior cranial view). The collateral artery in this case grew only after the mid LAD occlusion with anterior wall myocardial infarction, thus providing no myocardial-salvaging effect.

**Fig. (8):**
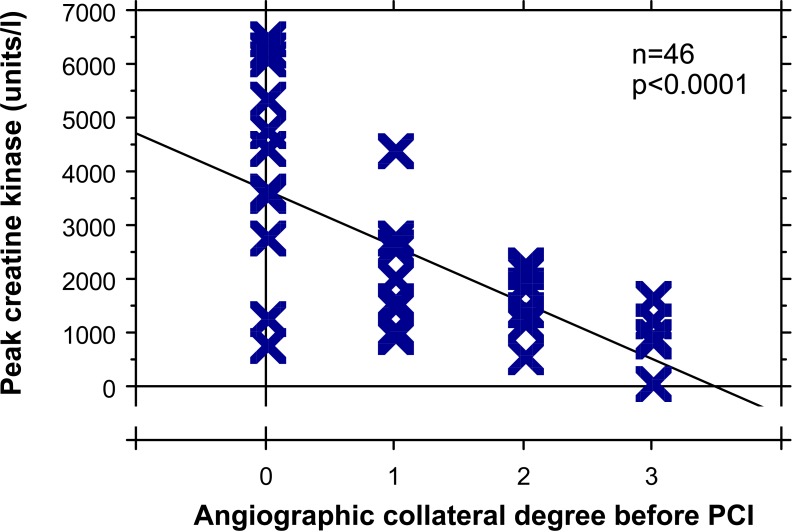
Scatter plot of peak creatine kinase (vertical axis) in patients with a first acute myocardial infarction and the angiographic collateral degree to the occluded vessel (horizontal axis) before primary percutaneous coronary intervention (PCI).

In general, beneficial effects of well functioning collaterals relating to myocardial salvage outweigh potential negative aspects as outlined below. In the individual patient, myocardial salvage by collaterals is very likely in the presence of normal left ventricular (LV) systolic function despite the total occlusion of ≥1 coronary arteries. In particular, this is the case in the presence of a proximal coronary occlusion. (Fig. **3**) illustrates the case of an elderly patient with normal systolic LV function despite proximal total occlusion of all three major coronary arteries. It may be challenging in this patient to define the principal collateral supply to each one of the occluded arteries. However, the presence of a normal LV systolic function allows to conclude that collateral supply at the time of occlusion was sufficient to salvage myocardium. (Fig. **4**) depicts the case of a 72 years old male patient with sufficiently collateralized LV anterior wall despite proximal left anterior descending (LAD) artery occlusion. A single atrial collateral artery originating from the ostial right coronary artery (RCA) supplies enough flow to entirely replace the LAD area at risk for infarction, i.e., to reduce it to zero. Even prior to RCA filling with contrast medium, the LAD is supplied by the proximal natural anastomosis, thus illustrating the concept of an inverse relation between collateral flow and area at risk for infarction [14]. The normal systolic function of the LV anterior wall in this patient allows the conclusion that the determinants of infarct size other than collateral supply played no role at the time of vascular occlusion because of the replacement of the LAD area at risk by one branch collateral artery [14]. A second, more proximal collateral vessel originating from the RCA appears to be even in competition with the main collateral vessel, because of the presence of a counter-flow coming from a proximal septal collateral artery (Fig. **4**). Another example of an individual collateral benefit is illustrated by (Fig. **5**). The 55 year old female patient was admitted for invasive cardiac examination in the context of atypical chest pain. Apart from the normal systolic LV function, the absence of exercise-induced angina pectoris already indicates the beneficial effect of the very large distal epicardial branch collateral artery from the RCA to the LAD (Fig **5**). On the other hand, diminished collateral filling of a chronic mid-LAD occlusion is related to extended LV anterior wall akinesia (Fig. **6**). Alternatively, extended collateral supply may grow only *after* coronary artery occlusion purely on physical grounds of an existing pressure drop between the collateral supplying and receiving artery, thereby providing an already infarcted myocardial territory (Fig. **7**). However if during acute myocardial infarction, there is already backward filling with contrast medium of the occluded coronary artery via collaterals, the subsequently developing infarct size is smaller than in the situation without preformed collaterals (Fig. **8**) [15].

## POTENTIAL NEGATIVE EFFECTS OF CORONARY COLLATERALS

**Fig. (9):**
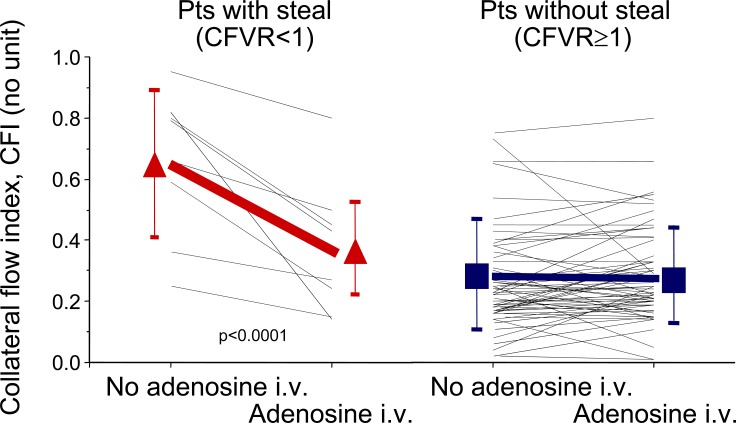
Collateral flow index values in 100 patients with coronary artery disease at rest and during adenosine infusion (140mcg/min) according to the presence or absence of intracoronary Doppler-derived coronary flow velocity reserve <1 (steal) in the collateral receiving, non-occluded artery. Symbols and error lines: mean and standard deviation.

**Fig. (10):**
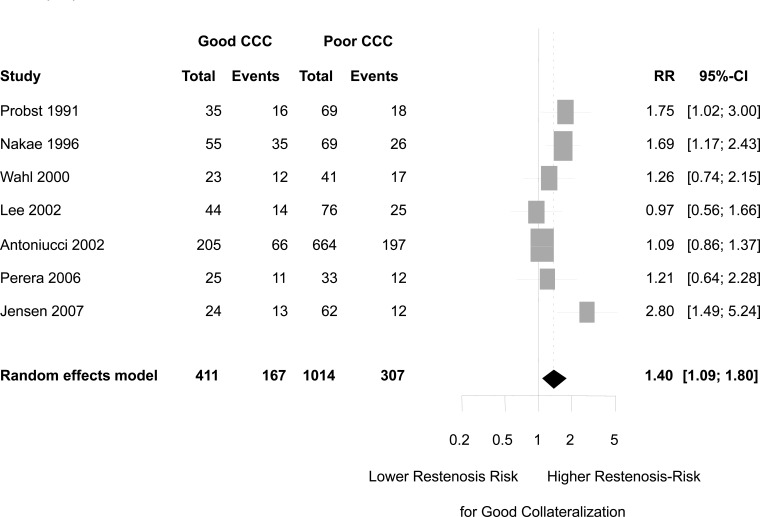
Forest plot of risk ratios (RR) for restenosis (≥ 50‰ diameter stenosis). CCC: Coronary collateral circulation. CI: confidence interval. Markers represent point estimates of risk ratios, marker size represents study weight in random-effects meta-analysis. Horizontal bars indicate 95‰ confidence intervals.

Aside from their beneficial effect on ischemic myocardium, well developed coronary collaterals may manifest negative aspects, such as coronary steal during myocardial hyperaemia (Fig. **9**) [16], and the risk of re-stenosis after coronary angioplasty (Fig. **10**) [17]. The physical mechanism leading to re-stenosis in the presence of well developed collaterals is thought to be competition between antegrade and collateral flow leading to reduced flow velocity at the injured site of PCI with augmented platelet adherence, thrombus formation, endothelial proliferation and developing re-stenosis.

**Fig. (11):**
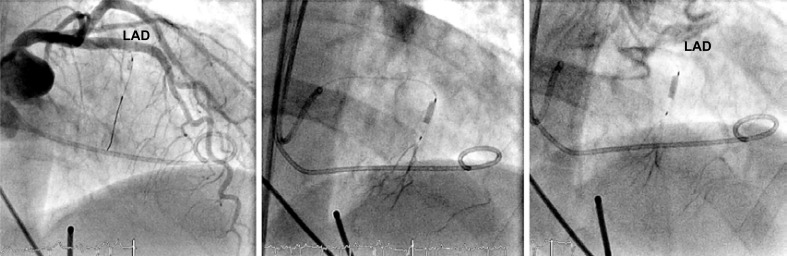
Transcoronary alcohol abalation of a 2nd septal branch (antero-posterior cranial view) in a patient with hypertrophic obstructive cardiomyopathy. During injection of contrast before alcohol injection (middle panel), the septal branch distal of the occluded balloon but also a more distal septal branch of the left anterior descending artery (LAD) is visualized via inter-coronary anastomoses. In the context of the subsequent injection of a small amount of alcohol, an acute occlusion of the LAD occurred (right panel; LAD).

An insufficiently recognized problem of septal alcohol ablation in hypertrophic cardiomyopathy is that of a functional collateral circulation even in the absence of CAD among 25% of individuals [12], in whom the alcohol injected into a septal branch can reach the RCA or LAD [18]. In the patient shown in (Fig. **11**), a preformed collateral artery between the balloon-occluded first and the third septal branch of the LAD was responsible for the adverse outcome of complete LAD occlusion following injection of 2.5ml 96-% alcohol over 11 minutes. Subsequently, this 60 year old female patient with initially severe LV outflow tract obstruction developed ventricular septal defect due to anterior wall myocardial infarction requiring emergency surgical intervention.

## COLLECTIVE PROGNOSTIC RELEVANCE

Though the coronary collateral circulation has long been recognized as an alternative source of blood supply to ischemic myocardium, its prognostic significance for the population of patients with CAD has been controversial until recently [19, 20]. The debate was in part related to different states of disease examined (acute versus chronic CAD, varying severity of CAD), to the imprecise and variable definition of the term “prognosis”, and to methodological issues such as the rare occurrence of study endpoints, the insufficient duration of follow-up and the blunt instrument employed to measure collateral supply. For example, two recent studies have documented a reduction in non-fatal cardiovascular events among patients with versus those without angiographic coronary collaterals in chronic stable CAD [21, 22]. On the other hand, data from the same group have indicated an unfavourable prognosis in the presence of angiographic collaterals among patients with more severe chronic CAD [23].

## ACUTE CORONARY ARTERY DISEASE AND CLINICAL EVENTS IN RELATION TO COLLATERALS

In a study by Waldecker *et al.* [24], angiographic collaterals to myocardium distal to an occluded coronary artery were detected in 334 of 626 patients during the acute infarct phase, whereby the prevalence was shown to increase between 3 and 6 hours following symptom onset (from 66 to 75%), and the absence of collaterals was associated with early cardiogenic shock among patients with inferior myocardial infarction [24]. Other investigations have observed collateral vessels at the beginning of acute myocardial infarction less often, i.e., in about 40% of patients [25, 26]. Schwartz *et al*. reported an analysis of the coronary collateral circulation in a series of 116 post-infarction angiograms from patients with persistent total occlusion of their infarct artery [26]. Of 42 patients studied within 6 hours of infarction, 52% had no angiographic evidence of collateral development as compared with only 8% studied 1-14 days after infarction [26]. Collaterals developing late after acute infarct into an area of necrotic myocardial tissue may exert a beneficial effect against LV remodelling, but not on LV systolic function [27]. On the other hand, residual blood flow carried by collaterals at the time of acute myocardial infarction implies reduced infarct size with improved LV ejection fraction [28, 29]. However, the question of whether collateral circulation improves clinical prognosis after acute myocardial infarction has not been frequently investigated and the answer seems to be still debatable [30-32]. In the context of the numerous determinants of collateral supply in acute coronary syndrome, such as the time window of study inclusion after symptom onset, the mode of revascularization (none, thrombolysis, PCI), the differentiation of preformed or subsequently grown collaterals, the mode of collateral assessment, the controversy is not unexpected.

### Studies in the Pre-angioplasty era

**Fig. (12):**
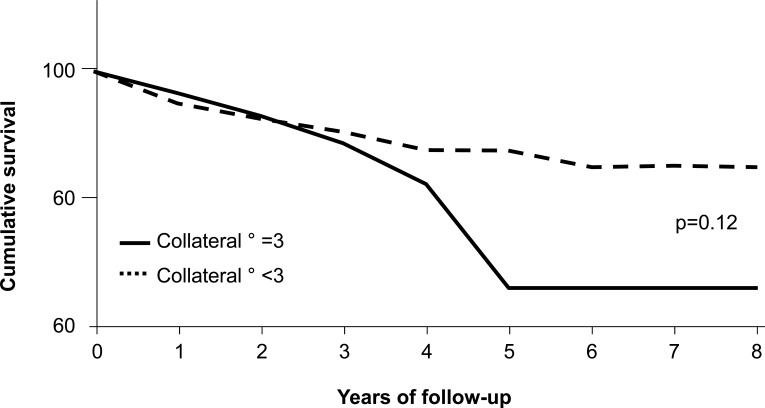
Cumulative survival curves in patients with acute myocardial infarction undergoing thrombolysis according to the presence (solid line) or absence (dashed line) of well grown collaterals to the infarct-related coronary artery as evidenced by angiography.

Gohlke *et al*. studied the prognostic relevance of a residual stenosis of the infarct artery and of collateral flow to the infarct zone in a group of 102 young patients who had survived a an acute transmural anteriormyocardial infarction [33]. Patients with vs those without collateral flow had a higher mortality rate of 21% vs 8%(p <0.05). This finding may not have reflected an absent protective effect of collaterals as the authors concluded, but it may have indicated the relevance of collaterals as a marker for coronary stenosis severity. Thus in that study, the actual prognostic comparison was primarily between patients with different degrees of residual LAD stenoses and not between different degrees of collateral flow [33]. Similarly, the investigation by Nicolau *et al.* was not one examining the impact on prognosis of a well developed coronary collateral circulation at the time of acute myocardial infarction (n=422 treated with thrombolysis and followed for 8 years) (Fig. **12**), but the variable prognostic effect of successful and partly successful or unsuccessful thrombolysis (survival rates of 89% and 80%, respectively; p<0.04) [34]. By Cox multivariate analysis in that study, the following *independent* determinants of long-term survival were found: global LV ejection fraction (p=0.0003), antegrade flow degree (p=0.0006), collateral flow degree (negative correlation, p=0.0179), and medical treatment (negative correlation, p=0.0464). Using a more favourable study methodology, Boehrer *et al.* assessed the influence of collateral filling of the infarct artery on long-term morbidity and mortality among 146 surviving patients in whom the infarct artery remained occluded [35]. Of 120 patients with angiographic evidence of collaterals 16% died from cardiac causes during the average follow-up time of 42 months, while 19% did so in the group of 26 patients without collaterals [35].

### Studies in Patients Undergoing Primary PCI

**Fig. (13):**
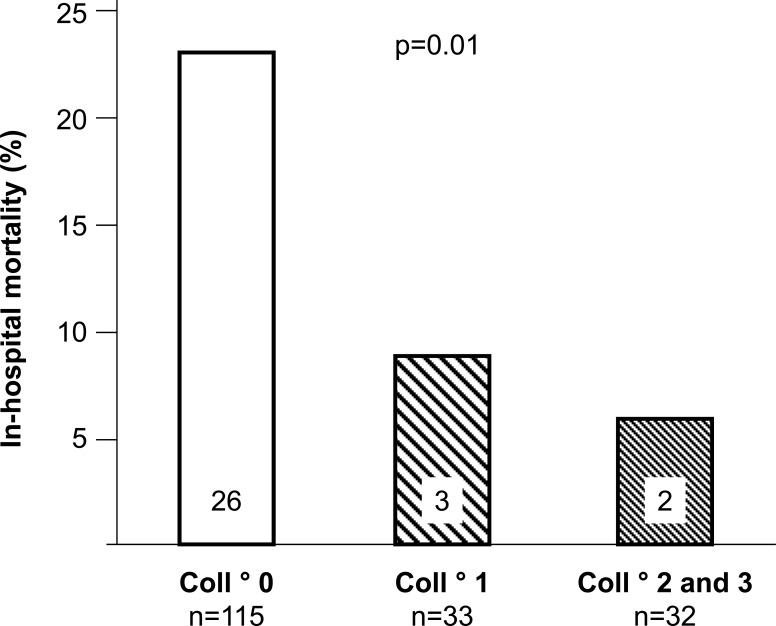
Box plot depicting in-hospital mortality following acute myocardial infarction (vertical axis) in relation to angiographic collateral degree (coll°; horizontal axis). Numbers in the boxes are absolute numbers of death.

**Fig. (14):**
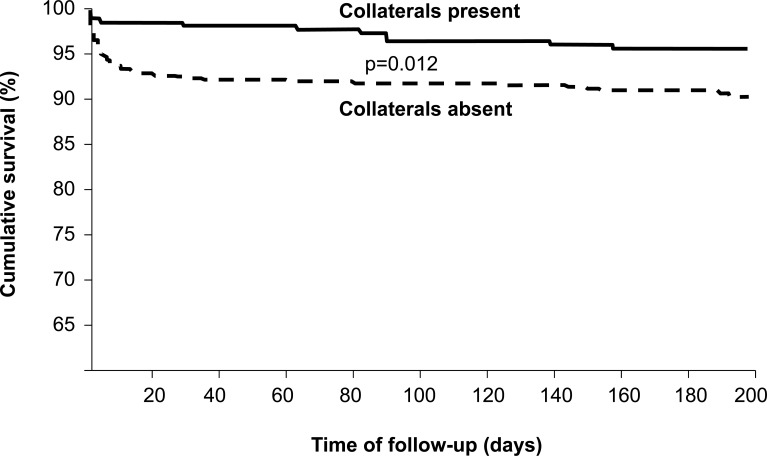
Cumulative survival curves of 264 patients with acute myocardial infarction and angiographic evidence of coronary collaterals supplying the infarct area (collaterals present; solid line), as compared to 900 patients without collaterals (collaterals absent; dashed line).

Studies on the prognostic impact of collaterals in patients undergoing successful primary PCI 6 hours of symptom onset are methodologically advantageous over the pre-PCI studies described above, because their populations are more homogeneous and therefore, better comparable. They are, however, rare and the results seem to be controversial. In an investigation including 238 patients with acute anterior myocardial infarction, Pérez-Castellano and colleagues found a significantly lower in-hospital mortality among patients with as compared to those without collaterals on the angiogram as obtained during primary PCI (Fig. **13**) [31]. On first sight and compared to the above cited study by Boehrer [35], that by Pérez-Castellano *et al*. appears similarly underpowered (n=146 versus n=180; absolute number of deaths: n=23 versus n=31). The principle difference consists of the varying number of patients with angiographically absent collaterals: 26/146 (18%) in the study by Boehrer *et al*. and 115/180 (64%) in that by Pérez-Castellano [31, 35]. This difference highlights the impact of the time course of collateral development following acute myocardial infarction on their prognostic influence: late angiographic appearance as in the study by Boehrer *et al*. is more frequent than early manifestation, but it means less prognostic benefit, because collaterals supplying necrotic myocardium do not salvage it. By study design, Pérez-Castellano *et al*. focused on collaterals present during the 6-hour time window after symptom onset, and thus, examined the relevance of preformed, i.e., more beneficial collaterals [31]. The study by Antoniucci *et al.* [30] is similar to that by Pérez-Castellano and colleagues [31], because both studied the outcome in patients with acute myocardial infaction undergoing primary PCI within 6 hours from symptom onset; the frequency of angiographic collaterals in both was similar, i.e., 23% respectively 36%. However, Antoniucci *et al*.’s work recruited a much larger population of 1’164 patients with acute infarct of any territory, and clinical events were registered until 6 months after revascularization [30]. At 6 months, 11/264 patients in the group with collaterals on angiography (4%) and 80/900 patients without collaterals (9%) had died (Fig. **14**) [30]. Due to an erroneous inclusion of clinical variables in the multivariate logistic regression model, the authors concluded that the coronary collateral circulation was not protective in patients undergoing revascularization within the first 6 hours of acute myocardial infarction [30]. The variables falsely entered in the statistical model were factors not different in univariate analysis between patients with and without collaterals (age, gender, previous myocardial infarction, PCI failure, infarct artery stenting, multiple stents), and factors not independent of each other (collateral circulation dependent on chronic total occlusion, on multivessel CAD). Thus, both studies on the prognostic impact of collaterals in acute myocardial infarction have reported a statistically relevant reduction in 6-month mortality from 9-23% to 4-7%. On the other hand, the study by Elsman *et al*. in 1’059 patients with acute ST-segment-elevation myocardial infarction treated by primary PCI within <6 hours of chest pain onset found cumulative 1-year survival rates not different between patients with angiographic collateral grades 0, 1 and 2 or 3: 95%, 96% and 97%, respectively (p=0.66) [36]. In the setting of subacute myocardial infarction, Steg *et al*. documented in 2’173 patients that angiographically present coronary collaterals were associated with lower cumulative 60-month event rates of death (p=0.009), heart failure (p=0.0001) or either one (p=0.0002), but had no association with the risk of reinfarction [32]. By multivariate analysis, collateral flow was neither an independent predictor of death nor of the primary trial composite end point of death, reinfarction, or class IV heart failure. 

In a recent meta-analysis on the topic [20], the risk ratio to die from any cause for high vs low or absent collateralization in patients with subacute myocardial infarction was 0.53 (95% confidence interval 0.15–1.92; p=0.335), and for patients with acute myocardial infarction, it was 0.63 (95% confidence interval 0.29–1.39; p=0.257).

## CHRONIC CORONARY ARTERY DISEASE AND CLINICAL EVENTS IN RELATION TO COLLATERALS

Regarding the dual role of the collateral circulation as a marker of CAD severity and a prognosticator of cardiac events, the setting of acute or chronic CAD is comparable. That is in both situations, an investigation focusing on the prognostic impact of collaterals should account for their role as indicators for CAD severity. In this context, the above study by Nicolau *et al*. of early infarct artery collateral flow after thrombolysis found conflicting results even within itself [34]: antegrade flow through the native, recanalized artery was observed to be directly, but collateral flow to be inversely related to survival. Biologically, this finding is unreasonable, because it is not relevant whether an ischemic region is supplied via native or via collateral vessels. While in the acute coronary syndrome, the collateral circulation as an indicator for CAD is corrected for by primary PCI, thus rendering the study population homogeneous for stenosis severity, a similar effect can be reached in chronic CAD by focusing exclusively on chronic total coronary occlusions. However, chronic total occlusion with downstream infarcted myocardium should be excluded from such an analysis.

### Angiographic Collateral Grading

**Fig. (15):**
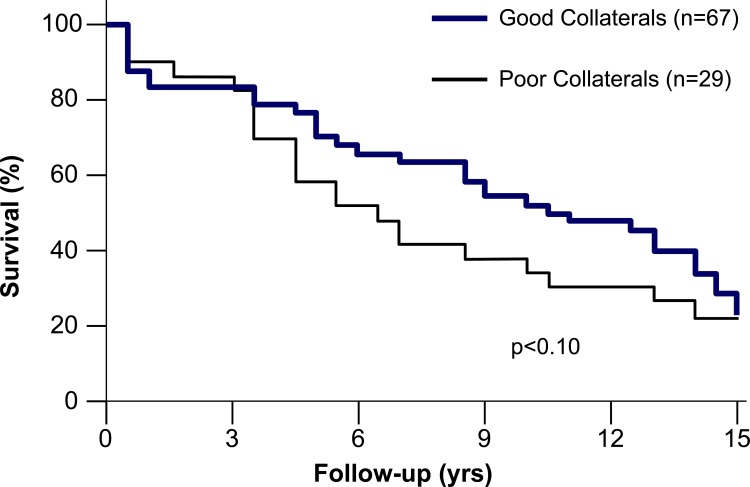
Cumulative survival curves in patients with chronic total coronary occlusion depending on the presence of angiographically qualified “good collaterals” (blue line) or “poor collaterals” (black line). None of the patients had had acute myocardial infarction within three months prior to coronary angiography. However, 29 of the patients with well grown collaterals and 23 of those with poorly developed collaterals had had a history of myocardial infarction.

The study by Hansen on the prognostic impact of collaterals in patients with chronic CAD did –in part- account for the described pitfall by restricting patient inclusion to those with occluded coronary arteries out of a series of 300 patients examined from 1968 to 1975 [37]. However, 52 of the 96 patients included in the study had suffered from myocardial infarction, whereby it isunclear how many had been in the region of interest. Of the 96 patients, 67 showed angiographically good and 29 poor collaterals. Ten-year cumulative survival rateswere 52% in patients with well developed collaterals and 35% among those with poor collaterals, respectively (Fig. **15**; p<0.10) [37]. The majority of studies on the prognostic influence of collaterals in chronic CAD have employed angiographic grading for collateral assessment. It was not until 2004 that the prognostic impact of coronary collaterals as assessed by angiographic collateral grading regained interest [21]. In 281 patients randomized to off-pump or on-pump coronary bypass surgery, Nathoe *et al*. found angiographic collaterals in close to 50% [21]. Cumulative 1-year event-free survival rates were 87% in patients with and 69% in those without collaterals after off-pump bypass surgery, respectively (p=0.01); the respective figures were 66% and 63%, respectively (p=0.79), following on-pump surgery [21]. The protective effect of collaterals in the off-pump group was related to fewer peri-operative myocardial infarcts than in the on-pump group. In an attempt to unravel the relevance of the collateral circulation as a marker of CAD severity from that as a prognosticator, Koerselman *et al*. carried out a case-control study in 244 patients admitted for elective PCI [23]. Angiographic collaterals were absent in 153 and present in 91 patients; the results indicated that in chronic CAD, the presence angiographically visible, but low grade collaterals were related to a prognostically adverse outcome [23]. The same research group studied the relation between angiographic collaterals and cardiac death or myocardial infarction at 1 year post-procedure in 561 patients who were enrolled in a randomized study comparing stenting with bypass grafting as the method of coronary revascularization [22]. Collaterals were visible in 176 patients (31%). The adjusted odds ratio of cardiac death or infarction was 0.18 (95% confidence interval 0.04 to 0.78) in the presence vs the absence of angiographically visible collaterals, and the cumulative 1-year event rate was 1.1% with and 5.3% without collaterals (p=0.01) [22]. The opposing conclusions regarding the prognostic impact of collaterals in the cited studies by Koerselman *et al*. [23] and by Nathoe *et al.* [22] are probably related to the low absolute number of cardiac deaths and myocardial infarctions. In comparison to these underpowered investigations, Abbott *et al*. compared the baseline characteristics and cumulative 1-year event rates with the target vessel collateral status in 6’183 patients undergoing PCI [38]. Collateral status was angiographically defined as absent (n=5’051), treated artery supplied collaterals to other vessels (n=239), and treated artery received collaterals from other vessels (n = 893). The major limitation of this study is the lack of a clear distinction between absent collaterals to the artery of interest and collaterals taking off that vessel. Such a distinction has to be regarded very difficult especially in the context of a data registry. Compared with the no-collaterals group, the collateral receiving target artery group had lower adjusted death/myocardial infarction rates (relative risk of 0.72, 95% CI 0.54 to 0.96, p = 0.02) and repeat revascularization rates (relative risk 0.73, 95% CI 0.59 to 0.91, p = 0.005) [38]. 

**Fig. (16):**
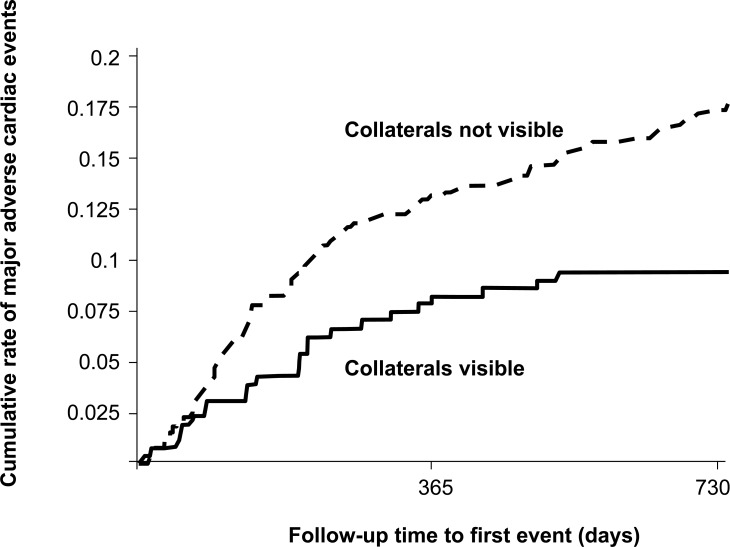
Cumulative event probability of major adverse cardiac events in patients with chronic coronary artery disease and angiographically visible (solid line) or invisible (dashed line) collateral vessels (p=0.002).

Recently, Regieli *et al.* retrospectively analyzed data from 879 male participants of a lipid lowering trial who underwent coronary angiography and were followed for 24 months [39]. The rate of spontaneously visible collaterals on angiography was assessed. Survival after two years free of cardiac death, infarct, repeat PCI and coronary artery bypass grafting was 84% in patients without collaterals, and 92% in patients with collaterals, respectively (p=0.0020; Fig. **16**). Though the absolute number of deaths and myocardial infarctions was low and similar to the abovementioned figures (8 deaths, 19 infarcts) [39], the authors concluded that the “protective effect is independent of disease burden, and remains present in patients with extensive CAD”.

### Quantitative Collateral Assessment

In a small cohort of 120 patients with chronic CAD undergoing PCI and followed for cardiovascular ischemic events for 6-22 months, Pijls and colleagues performed quantitative assessment of collateral relative to normal antegrade flow recruitable during a 2-minute vessel occlusion [40]. In 90 of the 120 patients, ischemia was present on ECG at balloon inflation, whereby in the majority of them (82/90) relative collateral flow was ≤23%. During follow-up, 16 patients died or suffered amyocardial infarction or unstable angina pectoris. Fifteen of these 16 patients were in the group with insufficient collateral flow (p<0.05) [40]. This study would be irrelevant regarding prognostic impact of collaterals in chronic CAD, because of its low statistical power. However, it illustrates that the introduction of a more precise measurement technique than angiography for collateral function assessment (coronary pressure derived relative collateral flow) can compensate for a small patient sample size and a brief follow-up duration.

**Fig. (17):**
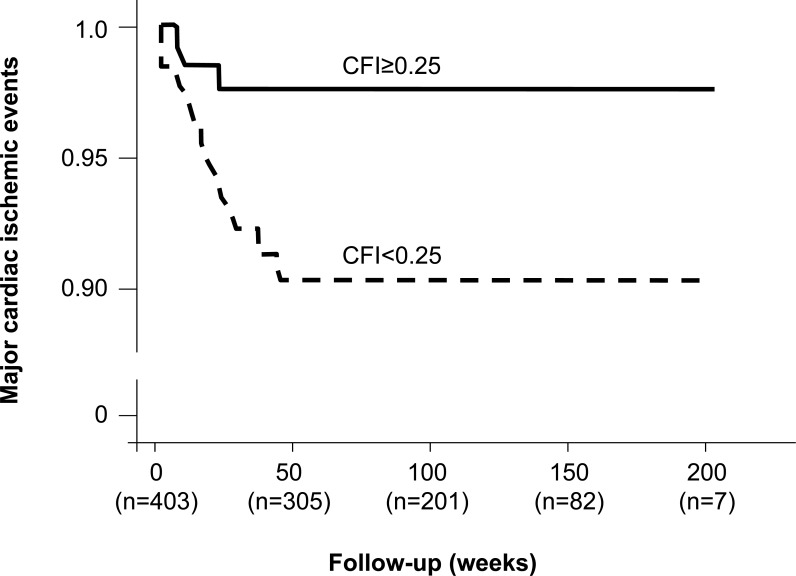
Cumulative event rate of death, myocardial infarction or unstable angina pectoris in 403 patients with chronic stable coronary artery disease according to the presence (solid line) or absence (dashed line) of collateral relative to normal antegrade flow (collateral flow index, CFI) ≥0.25.

In 403 patients with chronic stable CAD undergoing PCI and quantitative collateral assessment, our laboratory monitored the occurrence of major adverse cardiac events defined as cardiac death, myocardial infarction or unstable angina pectoris during an average follow-up of 94±56 weeks [41]. Relative collateral flow was measured using an intracoronary pressure or Doppler guidewire. The overall cardiac ischemic event rate during follow-up was 23% in patients with good collateral function (≥0.25 relative to normal flow) and 20% in patients with poor collateral function (p=not significant). However, only 2.2% of patients with a collateral flow index (CFI) ≥0.25 (see below for CFI calculation) suffered a major adverse cardiac ischemic event as compared to 9.0% among patients with CFI<0.25 (p=0.01; Fig. **17**). The incidence of stable angina pectoris was higher in patients with well than in those with poorly developed collaterals (21% vs. 12%; p=0.01) [41], thus suggesting the relevance of sufficient collaterals as marker for CAD severity in vessels not treated by PCI. The finding of a lowered cardiac event rate suggests their relevance as prognostic factor. However as several other studies cited in this paragraph, the one by Billinger *et al*. was also underpowered to allow answering the question whether collaterals are lifesaving in patients with chronic CAD [41]. 

**Fig. (18):**
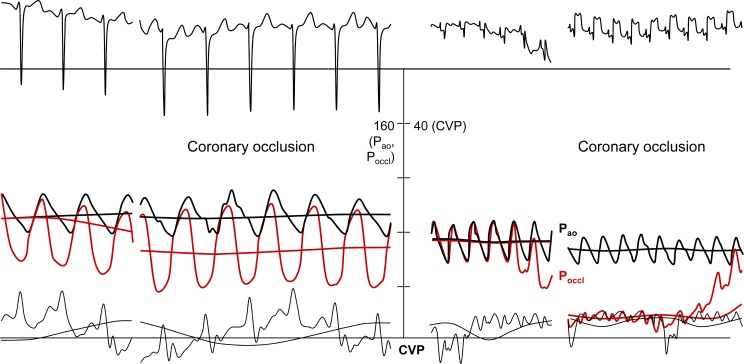
Intracoronary ECG (upper part) and pressure tracings (lower part) in a patient without evidence of myocardial ischemia during a 1-minute coronary balloon occlusion (left side; no ECG signs of ischemia, i.e., sufficient collaterals), and in a patient with marked ECG ST-segment elevations during the 1-minute coronary occlusion (right side; insufficient collaterals). Concomitantly, mean and phasic distal coronary pressure decreased much less during coronary occlusion (Poccl, red pressure curves) in the patient with sufficient collaterals than in the patient with insufficient collaterals (right side). Abbreviations: Pao: aortic pressure (black pressure curve); CVP: central venous pressure (black pressure curve).

**Fig. (19):**
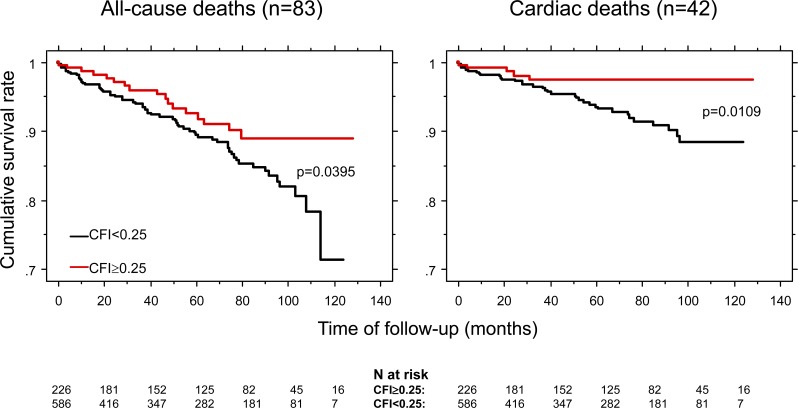
Cumulative survival rates in patients with chronic stable coronary artery disease related to all-cause (left side) and cardiac (right side) mortality according to the presence of low and high collateral flow index (CFI, i.e., collateral relative to normal antegrade coronary flow).

**Fig. (20):**
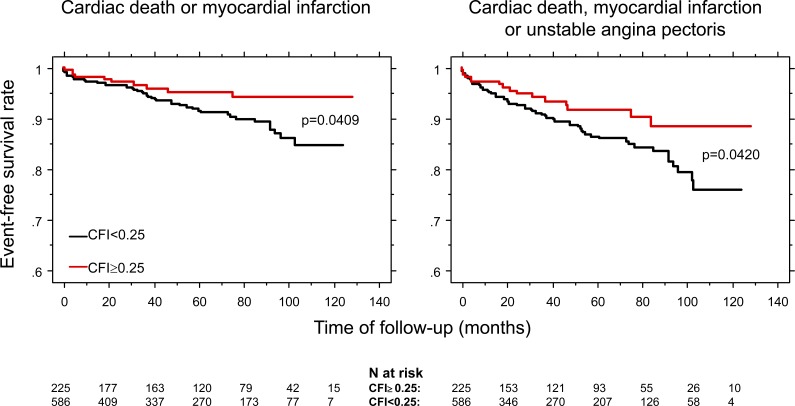
Cumulative event-free survival rates related to major adverse cardiac events: cardiac death or myocardial infarction (left side), cardiac death, myocardial infarction or unstable angina pectoris (right side) according to the presence of low and high collateral flow index (CFI, i.e., collateral relative to normal antegrade coronary flow).

The analysis of a database on quantitative functional collateral assessment (see below) has provided clinical and hemodynamic data of 845 individuals, 106 patients without CAD and 739 patients with chronic stable CAD. These individuals underwent a total of 1053 quantitative, coronary pressure-derived collateral measurements between March 1996 and April 2006 [19]. All of them have been prospectively included in the CFI database containing information on recruitable collateral function parameters obtained during a 1-minute coronary artery balloon occlusion. CFI was calculated as the ratio of mean distal coronary occlusive (Poccl) to aortic pressure (Pao) both subtracted by central venous pressure (CVP): CFI = (Poccl-CVP)/(Pao-CVP) (Fig. **18**). In this study by Meier P *et al*. [19], patients were divided into groups with poorly (CFI<0.25) or well functioning collateral vessels (CFI≥0.25). Information on the occurrence of all-cause mortality and major adverse cardiac events (cardiac death, infarct, unstable angina pectoris) after study inclusion was collected. Cumulative 10-year survival rates free of all-cause and cardiac deaths were 71% and 88%, respectively, in patients with low CFI, and 89% and 97% in the group with high CFI (p=0.0395, p=0.0109; Fig. **19**). The cumulative survival rate free of combined major adverse cardiac events is shown on (Fig. **20**). 

**Fig. (21):**
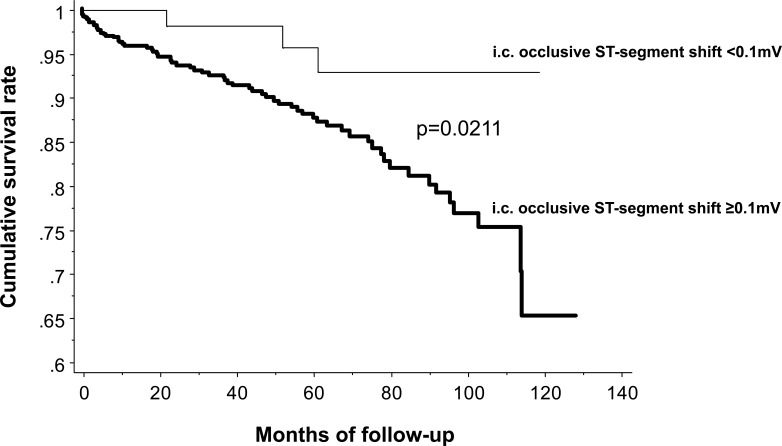
Cumulative event-free survival rates related to all-cause mortality (vertical axis) according to the presence of intracoronary (i.c.) ECG ST-segment shift amounting to <0.1mV (thin line) and ≥0.1mV (bold line) during a 1-minute coronary balloon occlusion.

Alternatively to the measurement of CFI, an intracoronary ECG taken from the angioplasty sensor guide wire was simultaneously obtained during coronary occlusion in all patients (Fig. **19**), that is, an alligator clamp was attached close to the end of the wire and connected to ECG lead V1. Thus, coronary collaterals without or with intracoronary ECG signs of ischemia (i.e. ST-segment elevation of < or ≥0.1mV) at the end of the 1-minute coronary occlusion were determined. Using this independent bivariate method for collateral assessment, cumulative 10-year survival free of all-cause mortality was 96% in patients with sufficient collaterals to prevent signs of ischemia during occlusion, and it was 65% among patients with insufficient collaterals, respectively (Fig. **21**) [42]. The following, statistically independent variables predicted elevated cardiac mortality by Cox proportional hazards analysis: age, low CFI (as continuous variable), current smoking [19].

**Fig. (22):**
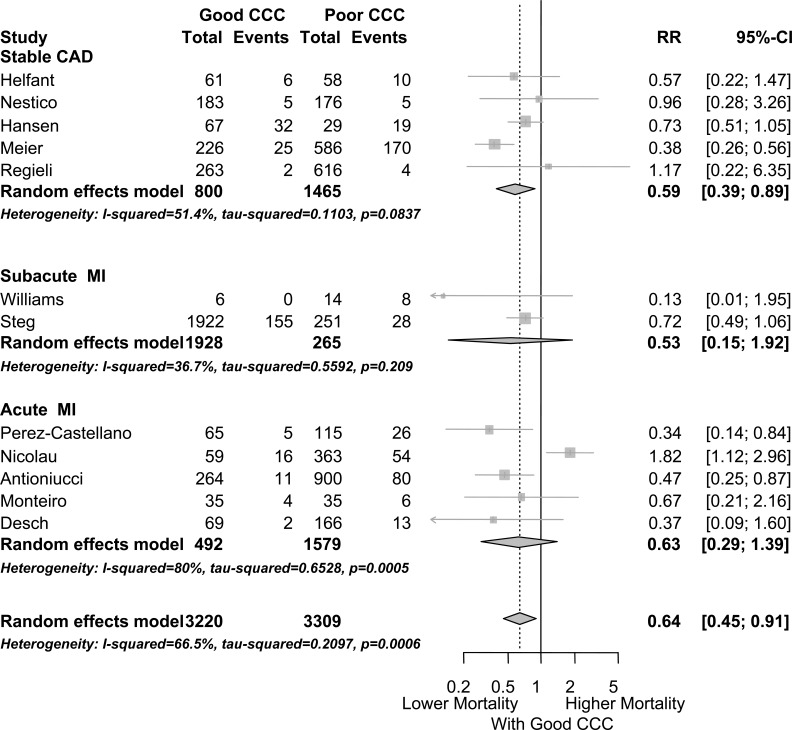
Forest plot of risk ratios (RR) for mortality risk, stratified by clinical setting (stable CAD, versus subacute MI, versus acute MI). CAD: coronary artery disease. CCC: Coronary collateral circulation. CI: confidence interval. MI: myocardial infarction. Horizontal bars indicate 95% confidence intervals.

In the above mentioned meta-analysis [20], the risk ratio to die from any cause for high vs low or absent collateralization in patients with stable CAD was 0.59 (95% confidence interval 0.39–0.89), p=0.012 (Fig. **22**).
